# Trends in pediatric prescription-opioid overdoses in U.S. emergency departments from 2008–2020: An epidemiologic study of pediatric opioid overdose ED visits

**DOI:** 10.1371/journal.pone.0299163

**Published:** 2024-04-17

**Authors:** Audrey Lu, Megan Armstrong, Robin Alexander, Eurella Vest, Jonathan Chang, Motao Zhu, Henry Xiang

**Affiliations:** 1 The Abigail Wexner Research Institute at Nationwide Children’s Hospital, Center for Pediatric Trauma Research, Columbus, OH, United States of America; 2 The Abigail Wexner Research Institute at Nationwide Children’s Hospital, Center for Injury Research and Policy, Columbus, OH, United States of America; 3 Biostatistics Resource at Nationwide Children’s Hospital (BRANCH), The Ohio State University, Columbus, OH, United States of America; 4 Ohio University Heritage College of Osteopathic Medicine, Dublin Campus, Dublin, OH, United States of America; 5 Department of Emergency Medicine, Nationwide Children’s Hospital, Columbus, OH, United States of America; 6 Department of Pediatrics, The Ohio State University College of Medicine, Columbus, OH, United States of America; Northeastern University, UNITED STATES

## Abstract

**Background:**

Opioid overdose was declared a public health emergency in the United States, but much of the focus has been on adults. Child and adolescent exposure and access to unused prescription-opioid medications is a big concern. More research is needed on the trend of pediatric (age 0–17) prescription-opioid overdose emergency department (ED) visits in the United States, particularly during the COVID-19 pandemic year.

**Methods:**

This retrospective epidemiological study used the 2008–2020 Nationwide Emergency Department Sample to provide a national estimate of ED visits related to prescription-opioid overdose. Inclusion criteria were 0-17-year-old patients treated at the ED due to prescription-opioid overdose. Eligible visits were identified if their medical records included any administrative billing codes for prescription-opioid overdose. National estimates were broken down by age groups, sex, geographic region, primary payer, median household income by zip code, ED disposition, and hospital location/teaching status. Incidence rate per 100,000 U.S. children was calculated for age groups, sex, and geographic region.

**Results:**

Overall, the prescription-opioid overdose ED visits for patients from 0–17 years old in the United States decreased by 22% from 2008 to 2019, then increased by 12% in 2020. Most patients were discharged to home following their ED visit; however, there was a 42% increase in patients admitted from 2019 to 2020. The prescription-opioid overdose rate per 100,000 U.S. children was highest in the 0 to 1 and 12 to 17 age groups, with the 12 to 17 group increasing by 27% in 2020. ED visits in the West and Midwest saw prescription-opioid visits increase by 58% and 20%, respectively, from 2019–2020.

**Conclusions:**

Prescription-opioid overdose ED visits among U.S. children and adolescents decreased over the past decade until 2019. However, there was a substantial increase in ED visits from 2019 to 2020, suggesting the potential impact due to the then-emerging COVID-19 pandemic. Findings suggest focusing on young children and adolescents to reduce further prescription-opioid overdoses in the United States.

## Introduction

Historical morbidity and mortality data suggest that opioid overdosing is a national crisis in the United States (U.S.), among both adults and children. In particular, between 1999–2016, nearly 9,000 children and adolescents died from opioid overdoses [1], while hospitalizations due to opioid poisoning increased from 1.4 to 3.7 per 100,000 children from 1997–2012 [2]. Suicidal poisonings due to opioid overdose among adolescents (15–19 years) also doubled, while unintentional poisonings increased three-fold. Opioids remain the source of most poisonings/overdoses in children under six years of age [2]. Children and adolescents are typically exposed to opioids in homes, schools, and communities [3–10]. This opioid exposure and overuse continue to be a major public health issue in the United States [11,12], leading the Department of Health and Human Services to declare the opioid epidemic a public health emergency in 2017 and again in 2022 [13].

Opioid use has increased since the 1990s, with a nine-fold increase in prescriptions in 2016 across the entire U.S. population [14]. Prior retrospective studies in children, adolescents, and young adults (ages 0–24 years) suggest that most opioids are obtained legally from physicians, with <10% of opioids purchased from the black market and other illegal channels [15]. However, over 65% of prescribed opioids go unused by the intended patients [16,17], making overprescribing a major concern. Overprescribing may be partly due to misleading marketing from pharmaceutical companies [18].

Due to the devastating impacts of opioid overconsumption, national and local guidelines were developed in an attempt to reduce the dose and frequency of opioid prescriptions [19,20]. The American Board of Internal Medicine board examination performance found that physicians with greater clinical knowledge of the potential harm of prescribed opioids were less likely to prescribe opioids than those with less clinical knowledge [21]. Many states developed prescription guidelines to prevent opioid overuse [17,22]. Some U.S. EDs have experimented with decreasing opioid prescribing to combat subsequent opioid misuse [23]. In addition, new approaches alerting physicians of opioid-related deaths of their patients have made physicians more conservative in their prescribing practices, including opioids, even if their prescription was irrelevant to the patient’s death [24]. Overall, efforts by researchers, public health professionals, and the news media have better informed the public about the potential negative consequences of opioid pain medication in general and the dangers associated with opioid overprescription specifically. These efforts are believed to have resulted in fewer opioid prescriptions since the early 2010s in the adult population [14].

Despite the nearly doubled risk of opioid dependency later in life after early exposure [15,25], the research conducted on opioid overdoses in children and adolescents is limited. Two studies have revealed an analogous relationship between the trends in opioid prescribing and hospitalizations for opioid-related poisoning in both adults and children [2,26]. Exposure of children and adolescents to opioids is often through family members’ leftover prescription medication [22], a major contributor to overdosing [15]. Another study found that opioid-related poisonings and adverse effects frequently occur secondary to a pediatric patient’s own prescription and without deviation from the prescribed regimen [27]. Low-income communities or patients in unstable social circumstances (like housing instability or parental divorce) are most at risk for drug overdosing [22].

This study examined patterns of pediatric prescription-opioid overdoses leading to ED visits from 2008 to 2020 using the Nationwide Emergency Department Sample (NEDS). Specifically, we evaluated the trends by patient and hospital characteristics to better understand potential contributors to prescription-opioid overuse in U.S. children and adolescent populations.

## Methods

### Data source

This retrospective, clinical, epidemiological study used 2008–2020 NEDS data sets [28]. NEDS is the largest all-payer ED database that provides national estimates of hospital-owned ED visits. NEDS is part of a family of databases and software tools produced by the Healthcare Cost and Utilization Project (HCUP), sponsored by the Agency for Healthcare Research and Quality. Specifically, the NEDS contains clinical and resource-use information in a typical patient discharge abstract without identifiable data to protect individual patient, physician, and hospital privacy.

To ensure a national representative sample, NEDS uses a 20% stratified sample of hospital-owned EDs in the United States [29]. Adjusting for this survey design and sampling weight, we calculated the national-level estimates of pediatric prescription-opioid overdose by year and the corresponding incidence rate per 100,000 children over the 12-year period. We also broke down the national-level estimates by demographic groups (see 2.2) to examine potential subpopulation patterns of prescription-opioid overdose patients. Due to the database being de-identified and publicly available, the Nationwide Children’s Hospital Institutional Review Board deemed this study exempt.

### Identifying opioid overdose cases and demographics

We used the International Classification of Diseases, Ninth Revision, Clinical Modification (ICD-9-CM), or International Classification of Diseases, Tenth Revision, Clinical Modification (ICD-10-CM) administrative billing codes to identify prescription-opioid overdose cases. In the NEDS, ICD-9-CM codes were used between quarter 1 of 2008 and the third quarter of 2015, including 96500, 96502, 96509, E8501, and E8502. ICD-10-CM codes were used from quarter four of 2015 through quarter four of 2020 and included T400X1-T400X4, T402X1-T402X4, T403X1-T403X4, T404X1-T404X2, T40601-T40604, and T40691-T40694. The major difference between the ICD-9-CM and ICD-10-CM is that ICD-10-CM contains more specific instructions for coding prescribed analgesics, especially opioids [24]. In this study, all diagnosis codes in the NEDS database (up to 35) were searched to identify cases of prescription-opioid overdose. Inclusion criteria were 0–17 years old patients treated at the ED due to prescription-opioid overdose.

The primary outcome of our study was characterizing pediatric prescription-opioid overdoses from 2008–2020. National estimates of pediatric prescription-opioid overdose were broken down by age in groups (0 to 1, 2 to 5, 6 to 11, 12 to 17, inclusive), sex (male or female), geographic region (Northeast, Midwest, South, West), primary payer (public, private, self-pay, other), median household income by zip code (2020 quartile 1 = $1–49,999; quartile 2 = $50,000–64,999; quartile 3 = $65,000–85,999; quartile 4 = $86,000+), ED disposition (routine, transfer to short term hospital, other transfers, admitted, home health care, died in ED, other), and hospital location/teaching status (rural, urban non-teaching, urban teaching) (Table 1).

**Table 1:** National estimates of pediatric prescription-opioid overdose by year and demographics.

	2008	2009	2010	2011	2012	2013	2014	2015	2016	2017	2018	2019	2020	% change 2008–2019	% change 2019–2020
**Total** National estimate	5874	5520	5803	6038	5697	5197	5521	4682	6794	6240	5625	4567	5124	-22%	12%
95% CI	5302, 6446	5014, 6026	5222, 6385	5378, 6699	5154, 6239	4618, 5776	4997, 6045	4257, 5106	6077, 7511	5579, 6901	4893, 6357	4015, 5120	4469, 5778		
**Sex**															
Female	3077	3058	3068	3155	3025	2996	3256	2605	4012	3636	3116	2430	2385	-21%	-2%
Male	2797	2452	2736	2878	2672	2201	2265	2077	2782	2600	2509	2138	2728	-24%	28%
**Age**															
0-<2	1049	858	1034	1099	1057	930	979	840	1274	1059	1195	907	896	-14%	-1%
2-<6	1044	1075	1115	1131	1274	1122	1093	1043	1430	1414	1228	1012	846	-3%	-16%
6-<12	185	173	187	291	210	145	272	258	255	306	263	160	190	-14%	19%
12-<18	3592	3409	3461	3508	3152	2990	3168	2541	3819	3446	2913	2481	3167	-31%	28%
**Region**															
Northeast	774	567	688	747	696	520	714	534	808	676	584	576	448	-26%	-22%
Midwest	1376	1473	1188	1564	1411	1330	1413	1010	1704	1705	1421	923	1104	-33%	20%
South	2115	2258	2135	2467	2069	1823	1990	2008	2581	2195	2365	1902	1742	-10%	-8%
West	1608	1221	1793	1261	1521	1523	1404	1131	1701	1664	1255	1167	1830	-27%	57%
**Primary Payer**															
Public	2244	2317	2769	2818	2755	2707	2900	2339	3553	3316	3248	2633	2999	17%	14%
Private	2836	2502	2443	2392	2326	1850	1951	1853	2528	2385	1919	1564	1680	-45%	7%
Self-pay	510	452	334	501	369	335	408	294	404	368	298	238	336	-53%	41%
Other	262	241	243	305	238	300	256	192	298	144	155	128	103	-51%	-20%
**Household income**															
Quartile 1 (lowest)	1600	1472	1561	1505	1510	1498	1538	1161	1929	1839	1887	1329	1525	-17%	15%
Quartile 2	1625	1598	1527	1672	1490	1461	1659	1253	1802	1786	1525	1211	1437	-25%	19%
Quartile 3	1239	1327	1411	1530	1401	1256	1290	1219	1577	1465	1187	1136	1052	-8%	-7%
Quartile 4 (highest)	1251	1008	1177	1244	1172	842	983	955	1411	1092	966	823	1042	-34%	27%
**ED Disposition**															
Routine	3600	3433	3438	3605	3485	2959	3270	2926	3724	3399	3117	2406	2730	-33%	13%
Transfer to short term hospital	463	480	582	654	543	630	534	480	878	841	615	692	623	49%	-10%
Other transfers	467	384	511	461	577	573	723	625	808	821	775	628	590	34%	-6%
Admitted	1271	1132	1174	1244	1020	959	925	0**	1287	1080	1072	779	1106	-39%	42%
Home health care	0	*	*	*	*	21	16	12	13	16	*	0	*		
Died in ED	*	*	*	*	0	0	0	0	*	*	0	0	20		
Other	66	82	88	61	63	55	54	639	78	74	38	62	49	-6%	-21%
**Hospital location/ teaching status**															
Rural	1103	1120	962	1055	975	972	844	883	1153	1075	798	711	571	-36%	-20%
Urban non-teaching	2586	2281	2706	2560	2286	2044	1621	1412	1703	1527	952	895	793	-65%	-11%
Urban teaching	2186	2119	2135	2423	2436	2181	3056	2387	3938	3638	3876	2962	3759	35%	27%

CI, confidence intervals; ED, emergency department.

*Cell values 1–10 suppressed due to the Healthcare Cost and Utilization Project data use agreement.

** The ICD-9 to ICD-10 transition in 2015 may have caused a coding discrepancy in the NEDS.

Values from 2008 to 2014 were calculated by searching on International Classification of Diseases, Ninth Revision, Clinical Modification (ICD-9-CM) codes. Values for the years 2015 to 2020 were calculated by searching on the 2015 International Classification of Diseases, Tenth Revision, Clinical Modification (ICD-10-CM) codes.

### Statistical analysis

Survey data analysis techniques were adjusted for both survey design and sample weights to yield national estimates of prescription-opioid overdose among children and adolescents to account for the stratified sampling design in NEDS. Specifically, NEDS developed survey weights using the American Hospital Association data of more than 6,200 hospitals and healthcare systems throughout the United States as the standard. Weighted discharge levels were calculated by strata to expand the ED visits in the NEDS sample to represent the national ED visits [29].

Descriptive statistics, including national estimates, percent change (2008–2019 and 2019–2020), and overdose rates, characterized overdose patterns from 2008 to 2020. The percentage change calculations’ date ranges were chosen to observe a trend line (2008–2019) and identify changes during the COVID-19 pandemic (2019–2020). The Taylor series linearization method estimated the standard error and 95% confidence intervals (95% CI) of the national estimates. Incidence rate per 100,000 U.S. children was calculated for age groups, sex, and geographic region by using the population data from the U.S. Census [30].

In compliance with the HCUP data use agreement, result table cell values of 1–10 were suppressed to avoid individual identification. SAS software version 9.4 (SAS Institute, Cary, NC) computed weighted counts and 95% CI [23] and ggplot2, an open-source data visualization package in R v1.3, plotted all figures [31–34].

## Results

The estimated national pediatric (0–17 years of age) prescription-opioid overdose cases are summarized by year (2008 to 2020) and demographic variables (Table 1). Despite some variability, overall, the prescription-opioid overdose ED visits in the United States decreased by 22% from 2008 to 2019, then increased by 12% in 2020. Patients with public or private insurance had higher case numbers than patients with self-pay or other insurance; however, in 2020, those with self-paid insurance saw an increase in ED visits by 47%. During most of the study period, lower-income households (quartiles 1 or 2 of the estimated median household income for the patient’s ZIP code) had more ED visits than higher-income households (Quartiles 3 and 4). In 2020, quartiles 1, 2, and 4 had increased ED visits for pediatric prescription-opioid overdose (15%, 19%, and 27%, respectively) compared to 2019. Most patients were discharged to home after an ED visit, followed by transfer and inpatient admission. However, a 42% increase in patients admitted was found from 2019 to 2020. Notably, cases of pediatric ED deaths were <10 annually between 2008–2019 but increased to 20 deaths in 2020. Urban teaching hospitals had the most ED visits, followed by urban non-teaching and rural hospitals. Prescription-opioid overdose ED visits increased by 27% in urban teaching hospitals from 2019–2020.

**Fig 1:**
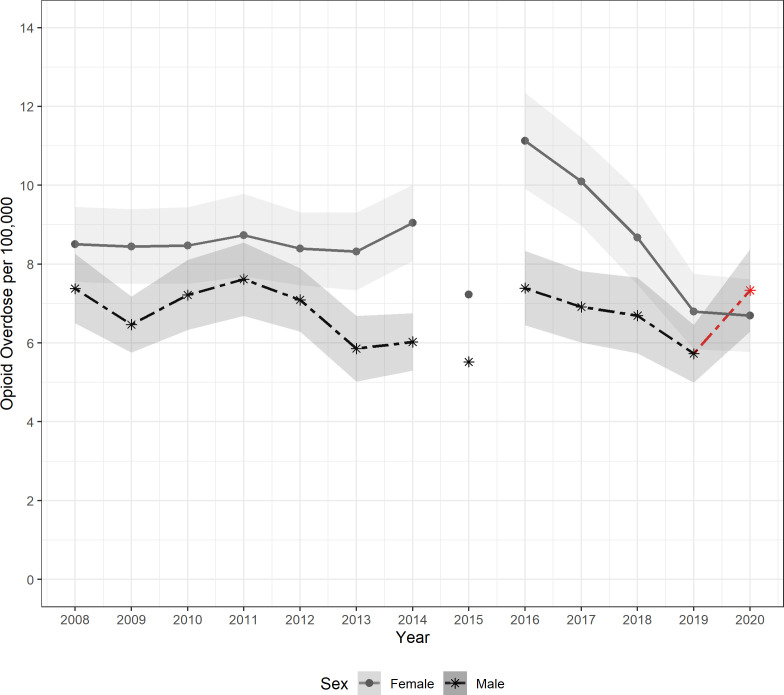
Pediatric prescription-opioid overdose rate by sex with 95% CI in shades of gray. The disconnected plot value in 2015 accounts for the 2015 transition from ICD-9-CM to ICD-10-CM and the change in case identification. Redline indicates an increase from 2019–2020.

Overall, males had fewer ED visits due to prescription-opioid overdose than females, except in 2020, when male ED visits increased by 28% compared to 2019, while female ED visits were stable. From 2008 through 2012, overdose rates were similar among both sexes, with overlap of the 95% confidence intervals each year, except in 2009. Both overdose rates stayed relatively constant during that period. From 2013, the disparity grew as female overdose rates increased overall between 2012 to 2014, but male overdose rates decreased. Data from 2015 was not used, and the disparity in 2016 was the largest over the entire study period with both male and female overdose rates being higher than they were in 2014. Part of the increased discrepancy may have been due to the implementation of the ICD-10-CM code in 2015. Overdose rates decreased for both sexes from 2016 to 2019, and so did the difference between the two groups. This trend was interrupted in 2020, when male overdose rates increased while female rates stayed stable, marking the only year during the study where male overdose rates were greater than female overdose rates. The largest difference between the two groups was 1,230 visits in 2016, the smallest difference was 277 in 2011, and the difference was 343 in 2020 (Fig 1). Overall, the rate of female prescription-opioid overdoses decreased from 2016 through 2020 by 40%.

**Fig 2:**
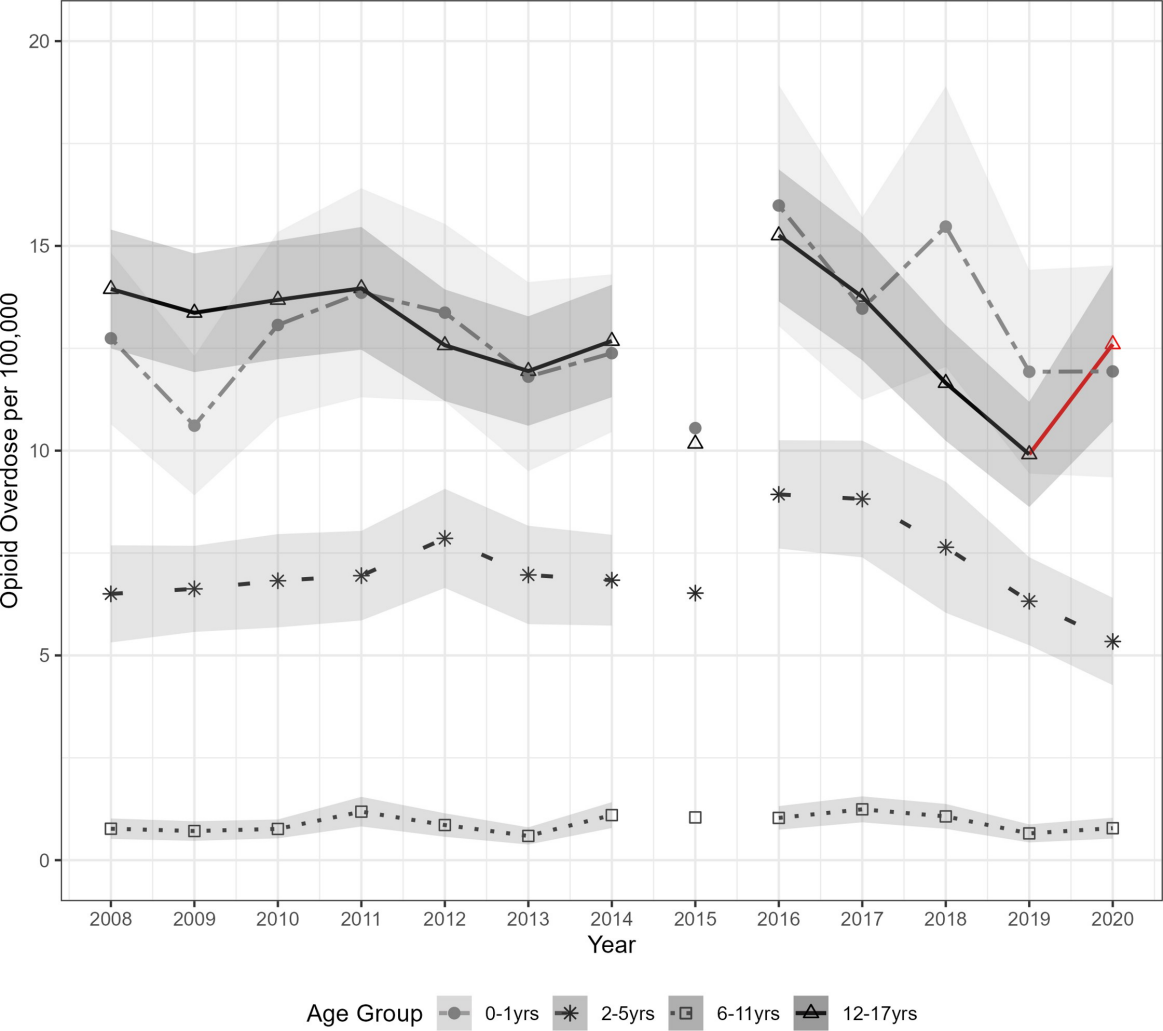
Pediatric prescription-opioid overdose rate by age group with 95% confidence intervals in shades of gray. The disconnected plot value in 2015 accounts for the 2015 transition from ICD-9-CM to ICD-10-CM and the change in case identification. Redline indicates an increase from 2019–2020.

While the 12 to 17 age group had the highest overall case numbers across the study period, the 6 to 11 and 12 to 17 age groups increased by 19% and 28%, respectively, from 2019 to 2020 (Table 1). The prescription-opioid overdose rate per 100,000 was highest in the 0 to 1 and 12 to 17 age groups (Fig 2). However, the 12 to 17 group increased by 27% in 2020, surpassing the 0 to 1 group. In the 6 to 11 group, rates of prescription-opioid overdose ED visits were consistent across the study period.

**Fig 3:**
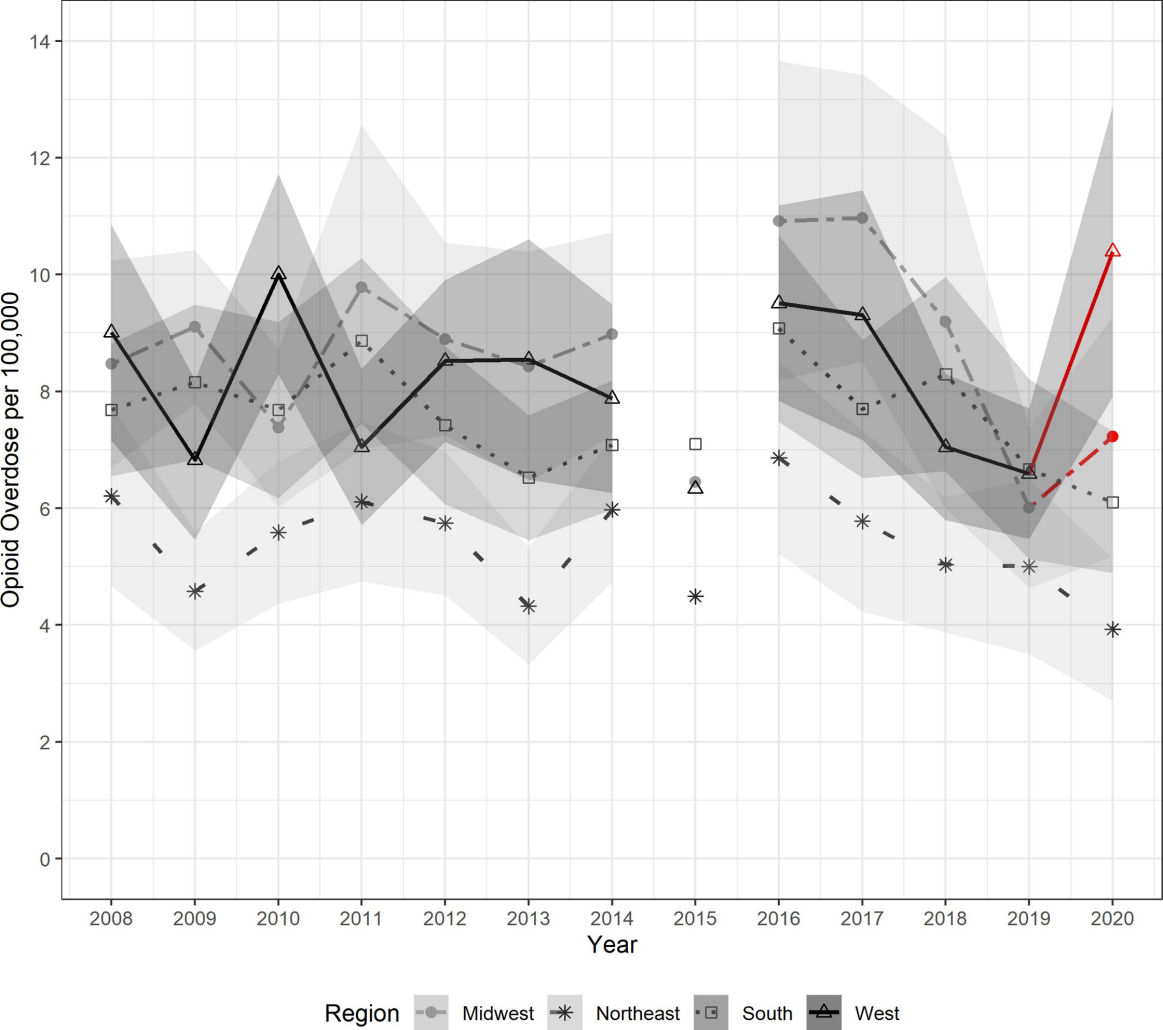
Pediatric prescription-opioid overdose rate by hospital region in the United States with 95% confidence intervals in shades of gray. The disconnected plot value in 2015 accounts for the 2015 transition from ICD-9-CM to ICD-10-CM and the change in case identification. Redline indicates an increase from 2019–2020.

Overall, ED visits were highest in the South, followed by the West, Midwest, and Northeast regions. In 2020, the West and Midwest increased ED visits by 57% and 20%, respectively, compared to 2019. When adjusted for population size, the Midwest, South, and West had similar ED visit rates for pediatric prescription-opioid overdose (Fig 3). However, the West and Midwest saw prescription-opioid ED visits increase by 58% and 20%, respectively, from 2019–2020.

## Discussion

Our study aimed to assess prescription-opioid overdose in children and adolescents by examining pediatric ED visits from 2008–2020 and evaluating trends by patient and hospital characteristics using a national dataset. We observed an overall decrease in prescription-opioid ED visits from 2008 to 2019 and an increase in prescription-opioid overdose ED visits from 2019 to 2020. Males, 12 to 17 age group, and those in the West and Midwest exhibited a large increase in prescription-opioid ED visits from 2019–2020.

Many governmental guidelines and interventions have increased physicians’ and patients’/parents’ awareness of the potential harm of prescription-opioid pain medications [17,19,20,22]. Previous studies have found higher overall adolescent drug use, often associated with more dangerous behaviors, mental instability, serious accidents, and higher death rates [11,35,36]. We analyzed for prescription-opioid overdose ED visits within four age groups (0 to 1, 2 to 5, 6 to 11, and 12 to 17 years). We also found that adolescents 12 to 17 had the most ED visits. Furthermore, the 0 to 1 years group had prescription-opioid ED visit frequencies comparable to the 12 to 17 years group, which ED physicians at our hospital have ascribed to young children unintentionally accessing caregiver medications [personal communication].

Data from this study suggested that females generally had more prescription-opioid overdose ED visits than males. However, prescription-opioid overdose ED visits for males increased by 28% from 2019 to 2020, with little change noted in females. Previous studies have indicated that females have a higher risk of opioid overdose than males [37,38], but these study populations did not focus on children or adolescents under 18. Bagley et al. found significant risk factors for prescription-opioid misuse among adolescents and young adults differed by sex, specifically comorbid psychiatric disorders (such as mood or anxiety disorders) and history of suicide attempts being higher in females than males [39].

The large decrease in 2015 and subsequent increase in 2016 could result from transitioning to using ICD-9-CM to ICD-10-CM codes in October 2015, which introduced notable changes in specificity for medical billing codes. This increased specificity could have improved the coding and identification of prescription-opioid overdoses that did not reflect an increase in the actual number of overdoses. Other studies in adult and geriatric populations have also observed a period of increased ED visits due to opioid overuse from 2015 to 2016 and an increase in 2020 [40]. Another possible factor would be the increased availability of synthetic opioids, such as illicitly manufactured fentanyl, and their contribution to the third and fourth wave of the opioid epidemic in the United States [41]. This increase in 2020 may be due to the rise in mental health concerns during the COVID-19 pandemic, which might have led to drug use in isolation and higher death rates [20] compared to <10 deaths from 2008–2019 [42,43].

There has been concern that decreasing opioid prescriptions will reduce physicians‘ ability to manage patients‘ pain effectively. Alternative medications include non-opioid analgesics, antidepressants, and anti-seizure drugs that have demonstrated pain-reducing benefits and lower risk of harm [44]. Nonpharmacologic pain treatments, such as exercise therapy and cognitive behavioral therapy, and medical virtual reality have been shown to be associated with the reduction of chronic pain [44–47]. Therefore, physicians should also consider non-opioid medications and nonpharmacologic pain strategies for pain management. When opioids are prescribed, our findings show that certain sociodemographic factors increase the likelihood of opioid overuse. Therefore, physicians should account for each patient’s circumstances to tailor the treatment plan to the individual, instead of standardizing prescriptions for all patients. Furthermore, medical staff should actively follow-up with patients to assess for signs of opioid misuse and gradually reduce opioid consumption.

The year of 2020 revealed many increases in opioid overdose inconsistent with the steady decrease observed from the years 2016–2019. This was most likely due to the outbreak of the COVID-19 pandemic. Due to the pandemic, a series of lockdowns were put in place. The World Health Organization (WHO) reports that depression rates increase by 25% during the pandemic [48], which has been historically associated with increased drug use. In addition, people who had already developed an opioid dependence would be cut off from their supply of prescription opioids at the hospital, so they would be more likely to turn to unreliable sources to obtain the drugs [49], such as dealers contacted on social media. These drugs would likely contain contaminants or have unknown concentrations of substance, causing harmful effects or increasing the potency of the opioids and therefore making it much easier to overdose. Finally, since people were confined to their homes, if a person overdosed, the chance that they would be discovered and quickly administered proper treatment was much lower [49], resulting the increased severity of many overdoses.

Limitations of this study should be noted. One limitation of our study is that the NEDS data sets contain records of ED encounters and subsequent discharges but do not identify patient characteristics, such as whether patients had more than one hospital ED visit for prescription-opioid poisonings. In addition, the ICD-9-CM and ICD-10-CM codes used in this study were originally coded for reimbursement purposes and not collected for research, which could be affected by hospital practice variations and overall institutional differences, likely leading to differences in data collection quality or underestimating prescription-opioid poisoning cases due to nonspecific coding practices [50]. Further, implementing the ICD-10-CM guidelines in late 2015 may have led to differences in overdose coding that could contribute to the appearance of frequency changes that did not occur [51]; however, our study included five years of data post the ICD-10-CM transition which minimizes the likelihood that the coding/billing guideline changes that occurred in 2015 will affect the generalizability of the study. Another limitation is that although the data on the total ED visits for pediatric prescription-opioid overdose was broken down by community type (such as household income and hospital location/teaching status) (Table 1), no data was available on the total number of children living in these areas to calculate rates. This study uses ED visits related to prescription opioid overdose as a proxy for U.S. prescription opioid overdose; however, it should be noted that the NEDS dataset only captures visits to the ED. This method underrepresents opioid overdoses that occur outside of a medical facility. Finally, race and ethnicity variables were not included in the NEDS data until 2019, so we could not evaluate these important variables in our study.

Future studies could investigate the impact of the COVID-19 pandemic on the opioid epidemic once nationwide data on opioid overdose is made available for later years of the pandemic. In particular, this data could be used to track further trends in opioid overdose for the sociodemographic subgroups of males, adolescents aged 12–17 years, and the Midwest and West regions that experienced large overdose rate increases in 2020.

## Conclusion

In the past decade, the number of prescription-opioid overdose ED visits has changed substantially for U.S. children and adolescents. Our study observed a 22% decrease in ED visits from 2008 to 2019, but 2019 to 2020 saw a 12% increase. We also found that children from 0 to 1 year and adolescents 12 to 17 years had the highest overall number and incidence rates of ED visits for prescription-opioid overdose compared to other age groups. Additionally, female children and adolescents generally had more prescription-opioid overdose ED visits than males. These study findings support focusing on young children (0 to 1 year), adolescents (12 to 17 years), and females to reduce further prescription-opioid overdoses in the United States.

## Supporting information

S1 Checklist STROBE statement—checklist of items that should be included in reports of observational studies. (DOCX)
