# Supplementary material for: Trends in pediatric prescription-opioid overdoses in U.S. emergency departments from 2008–2020: An epidemiologic study of pediatric opioid overdose ED visits
Source: PLoS One. 2024 Apr 17;19(4):e0299163. doi: 10.1371/journal.pone.0299163 (PMC11023208; doi:10.1371/journal.pone.0299163)
Supplement: S1 Checklist — (DOCX) [file pone.0299163.s001.docx]

STROBE Statement—checklist of items that should be included in reports of observational studies

|  | Item No. | Recommendation | Page  No. | Relevant text from manuscript |
| --- | --- | --- | --- | --- |
| **Title and abstract** | 1 | (*a*) Indicate the study’s design with a commonly used term in the title or the abstract | 1 | Trends in pediatric prescription-opioid overdoses in U.S. emergency departments  from 2008-2020: and epidemiologic study |
|  |  | (*b*) Provide in the abstract an informative and balanced summary of what was done and what was found | 2-3 | See manuscript lines 42-65 |
| Introduction | | | |  |
| Background/rationale | 2 | Explain the scientific background and rationale for the investigation being reported | 4-5 | See manuscript lines 68-109 |
| Objectives | 3 | State specific objectives, including any prespecified hypotheses | 5-6 | This study examined patterns of pediatric prescription-opioid overdoses leading to ED visits from 2008 to 2020 using the Nationwide Emergency Department Sample (NEDS). Specifically, we evaluated the trends by patient and hospital characteristics to better understand potential contributors to prescription-opioid overuse in U.S. children and adolescent populations. |
| Methods | | | |  |
| Study design | 4 | Present key elements of study design early in the paper | 6 | This retrospective, clinical, epidemiological study used 2008-2020 NEDS data sets |
| Setting | 5 | Describe the setting, locations, and relevant dates, including periods of recruitment, exposure, follow-up, and data collection | 6 | See lines 116-122 |
| Participants | 6 | (*a*) *Cohort study*—Give the eligibility criteria, and the sources and methods of selection of participants. Describe methods of follow-up  *Case-control study*—Give the eligibility criteria, and the sources and methods of case ascertainment and control selection. Give the rationale for the choice of cases and controls  *Cross-sectional study*—Give the eligibility criteria, and the sources and methods of selection of participants | 6-7 | See lines 132-143 for identifying cases using ICD-9 and ICD-10 codes |
|  |  | (*b*) *Cohort study*—For matched studies, give matching criteria and number of exposed and unexposed  *Case-control study*—For matched studies, give matching criteria and the number of controls per case | N/A | N/A |
| Variables | 7 | Clearly define all outcomes, exposures, predictors, potential confounders, and effect modifiers. Give diagnostic criteria, if applicable | 7 | See lines 144-151 |
| Data sources/ measurement | 8* | For each variable of interest, give sources of data and details of methods of assessment (measurement). Describe comparability of assessment methods if there is more than one group | 7 | See lines 144-151 |
| Bias | 9 | Describe any efforts to address potential sources of bias | N/A | N/A |
| Study size | 10 | Explain how the study size was arrived at | N/A | N/A |

Continued on next page

| Quantitative variables | 11 | Explain how quantitative variables were handled in the analyses. If applicable, describe which groupings were chosen and why | 8 | See lines 160-170 |
| --- | --- | --- | --- | --- |
| Statistical methods | 12 | (*a*) Describe all statistical methods, including those used to control for confounding | 7-8 | See lines 153-170 |
|  |  | (*b*) Describe any methods used to examine subgroups and interactions | N/A | N/A |
|  |  | (*c*) Explain how missing data were addressed | N/A | N/A |
|  |  | (*d*) *Cohort study*—If applicable, explain how loss to follow-up was addressed  *Case-control study*—If applicable, explain how matching of cases and controls was addressed  *Cross-sectional study*—If applicable, describe analytical methods taking account of sampling strategy | 7-8 | See lines 153-159 |
|  |  | (*e*) Describe any sensitivity analyses | N/A | N/A |
| Results | | | | |
| Participants | 13* | (a) Report numbers of individuals at each stage of study—eg numbers potentially eligible, examined for eligibility, confirmed eligible, included in the study, completing follow-up, and analysed | N/A | N/A |
|  |  | (b) Give reasons for non-participation at each stage | N/A | N/A |
|  |  | (c) Consider use of a flow diagram | N/A | N/A |
| Descriptive data | 14* | (a) Give characteristics of study participants (eg demographic, clinical, social) and information on exposures and potential confounders | 10-12 | See Table 1 |
|  |  | (b) Indicate number of participants with missing data for each variable of interest | N/A | N/A |
|  |  | (c) *Cohort study*—Summarise follow-up time (eg, average and total amount) | N/A | N/A |
| Outcome data | 15* | *Cohort study*—Report numbers of outcome events or summary measures over time | N/A | N/A |
|  |  | *Case-control study—*Report numbers in each exposure category, or summary measures of exposure | N/A | N/A |
|  |  | *Cross-sectional study—*Report numbers of outcome events or summary measures | 10-12 | See Table 1 |
| Main results | 16 | (*a*) Give unadjusted estimates and, if applicable, confounder-adjusted estimates and their precision (eg, 95% confidence interval). Make clear which confounders were adjusted for and why they were included | 10-12 | See Table 1 |
|  |  | (*b*) Report category boundaries when continuous variables were categorized | 10-12 | See Table 1 |
|  |  | (*c*) If relevant, consider translating estimates of relative risk into absolute risk for a meaningful time period | N/A | N/A |

Continued on next page

| Other analyses | 17 | Report other analyses done—eg analyses of subgroups and interactions, and sensitivity analyses | 13 | See figures 1-3 |
| --- | --- | --- | --- | --- |
| Discussion | | | | |
| Key results | 18 | Summarise key results with reference to study objectives | 14-15 | See lines 224-255 |
| Limitations | 19 | Discuss limitations of the study, taking into account sources of potential bias or imprecision. Discuss both direction and magnitude of any potential bias | 15-16 | See lines 256-275 |
| Interpretation | 20 | Give a cautious overall interpretation of results considering objectives, limitations, multiplicity of analyses, results from similar studies, and other relevant evidence | 16 | See lines 277-284 |
| Generalisability | 21 | Discuss the generalisability (external validity) of the study results | 15 | Lines 263-267 |
| Other information | |  | | |
| Funding | 22 | Give the source of funding and the role of the funders for the present study and, if applicable, for the original study on which the present article is based | N/A | Submitted within PLOS ONE submission as per author guidelines |

*Give information separately for cases and controls in case-control studies and, if applicable, for exposed and unexposed groups in cohort and cross-sectional studies.

**Note:** An Explanation and Elaboration article discusses each checklist item and gives methodological background and published examples of transparent reporting. The STROBE checklist is best used in conjunction with this article (freely available on the Web sites of PLoS Medicine at http://www.plosmedicine.org/, Annals of Internal Medicine at http://www.annals.org/, and Epidemiology at http://www.epidem.com/). Information on the STROBE Initiative is available at www.strobe-statement.org.
